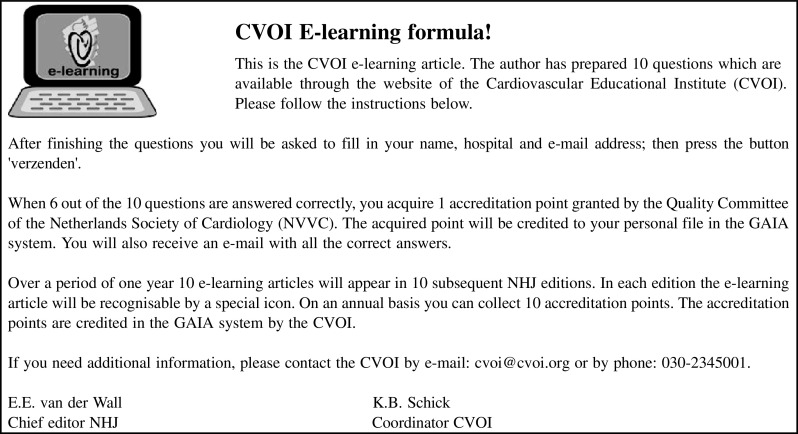# Assessment of left ventricular ejection fraction in patients eligible for ICD therapy: Discrepancy between cardiac magnetic resonance imaging and 2D echocardiography

**DOI:** 10.1007/s12471-014-0594-0

**Published:** 2014-09-04

**Authors:** S. de Haan, K. de Boer, J. Commandeur, A. M. Beek, A. C. van Rossum, C. P. Allaart

**Affiliations:** Department of Cardiology and Institute for Cardiovascular Research (ICaR-VU), VU University Medical Center, De Boelelaan 1118, 1081 HV Amsterdam, the Netherlands

**Keywords:** Cardiac magnetic resonance imaging, Echocardiography, Left ventricular ejection fraction

## Abstract

**Objective:**

Implantable cardioverter defibrillators (ICD) and cardiac resynchronisation therapy (CRT) have substantially improved the survival of patients with cardiomyopathy. Eligibility for this therapy requires a left ventricular ejection fraction (LVEF) <35 %. This is largely based on studies using echocardiography. Cardiac magnetic resonance imaging (CMR) is increasingly utilised for LVEF assessment, but several studies have shown differences between LVEF assessed by CMR and echocardiography. The present study compared LVEF assessment by CMR and echocardiography in a heart failure population and evaluated effects on eligibility for device therapy.

**Methods:**

152 patients (106 male, mean age 65.5 ± 9.9 years) referred for device therapy were included. During evaluation of eligibility they underwent both CMR and echocardiographic LVEF assessment. CMR volumes were computed from a stack of short-axis images. Echocardiographic volumes were computed using Simpson’s biplane method.

**Results:**

The study population demonstrated an underestimation of end-diastolic volume (EDV) and end-systolic volume (ESV) by echocardiography of 71 ± 53 ml (mean ± SD) and 70 ± 49 ml, respectively. This resulted in an overestimation of LVEF of 6.6 ± 8.3 % by echocardiography compared with CMR (echocardiographic LVEF 31.5 ± 8.7 % and CMR LVEF 24.9 ± 9.6 %). 28 % of patients had opposing outcomes of eligibility for cardiac device therapy depending on the imaging modality used.

**Conclusion:**

We found EDV and ESV to be underestimated by echocardiography, and LVEF assessed by CMR to be significantly smaller than by echocardiography. Applying an LVEF cut-off value of 35 %, CMR would significantly increase the number of patients eligible for device implantation. Therefore, LVEF cut-off values might need reassessment when using CMR.

## Introduction

Cardiac resynchronisation therapy (CRT) and the implantable cardioverter defibrillator (ICD) have substantially improved the survival of heart failure patients.[1–4] A key criterion for selection of CRT and ICD candidates is a severely depressed left ventricular ejection fraction (LVEF). In daily clinical practice, as well as in all large clinical trials, assessment of LVEF is mainly performed by two-dimensional (2D) echocardiography due to its wide availability and guidelines do not recommend a method for LVEF assessment.[1–4] Cardiac magnetic resonance imaging (CMR) is considered the gold standard to assess LVEF and is increasingly utilised for LVEF assessment in routine clinical settings.[5–10] Several reports have been published on the comparison between assessment by 2D echocardiography and CMR.[5, 11–14] These studies consistently reported a significant underestimation of both left ventricular end-diastolic and systolic volumes by 2D echocardiography. On calculation of LVEF, however, conflicting results were found. The majority of these studies were performed in patients with (near) normal LVEF and relatively small sample sizes were used. The consequences of these differences between the two imaging modalities on ejection fraction in heart failure patients and thus for eligibility for cardiac device therapy are largely unknown, although there are suggestions that CMR is preferable for LVEF assessment in the case of selection for device implantation.[15, 16] The present study compared LVEF assessed by 2D echocardiography and CMR in a large group of heart failure patients who were referred for evaluation of eligibility for device therapy and the consequences for eligibility were evaluated.

## Methods

### Study population

Patients referred to the VU University Medical Center for CRT and/or ICD implantation for primary prevention according to current guidelines and who underwent both 2D echocardiographic and CMR evaluation within 3 months prior to implantation were included. A total of 152 patients with chronic stable heart failure met these criteria. All patients were under optimal medical therapy and there was no change in medication or clinical condition between each assessment. All analyses were done according to daily clinical practice and both assessments are used regularly at our institution.

### CMR image acquisition and analysis

CMR studies were performed on a 1.5-Tesla whole body scanner (Magnetom Sonata/Avanto, Siemens, Erlangen, Germany), using a six-channel phased-array body coil. After survey scans, a retro-triggered, balanced steady-state free precession gradient-echo sequence was used for cine imaging. Image parameters included slice thickness of 5 mm, slice gap 5 mm, temporal resolution <50 ms, repetition time 3.2 ms, echo time 1.54 ms, flip angle 60° and a typical image resolution of 1.3*1.6 mm. Stacks of 10–12 short-axis slices were acquired to cover the left ventricle. Cine images were acquired during breath-hold in mild expiration.

Images were analysed off-line, using the software package MASS (MR Analytical Software System, Medis, Leiden, the Netherlands). Endocardial borders of the left ventricle were outlined manually in both the end-diastolic and end-systolic phase in all short-axis images. Papillary muscles were included in the left ventricular volume. End-diastolic volume (EDV), end-systolic volume (ESV), and LVEF were computed using these analyses as previously described.[17]

### Echocardiographic image acquisition and analysis

Two-dimensional echocardiographic images were obtained in the standard parasternal long- and short-axis and apical four-chamber and two-chamber views using commercially available ultrasound equipment. Three cardiac cycles were captured and no ultrasound contrast agents were used to enhance image quality. Echocardiographic measurements were performed offline from the apical windows by an expert reader blinded to patient data. Left ventricular volumes and LVEF were planimetred from the four-chamber and two-chamber areas using the modified Simpson’s rule. Papillary muscles were included in the left ventricular volumes and all measurements were done in concordance with the American Society of Echocardiography standards.[18]

### Interobserver and intraobserver variability

Measurements of the left ventricular volumes were repeated in 15 subjects by the same observer and an experienced second observer both blinded for previous measurements to assess interobserver and intraobserver variability.

### Statistical analysis

Continuous variables are presented as mean ± SD, and categorical data are summarised as frequencies and percentages. Comparisons between the imaging techniques were made using the Blant-Altman analysis. A linear regression analysis was performed on the Blant-Altman data to evaluate whether differences were dependent of volumes. Intraobserver and interobserver variability were assessed using the Blant-Altman analysis. A value of *p* < 0.05 was considered statistically significant. The statistical analysis was performed by means of SPSS for Windows (version 16.0, SPSS Inc., Chicago, USA).

## Results

A total of 152 patients with chronic stable heart failure were included. The aetiology of heart failure was ischaemic cardiomyopathy in 52 % and dilated cardiomyopathy in 48 % of patients. Time between CMR and 2D echocardiography assessment was a median of 7 days (interquartile range 0–35). Baseline characteristics are depicted in Table 1.

**Table 1:** Baseline characteristics

Baseline characteristics	*n* = 152
Age (years)	65.5 ± 9.9
Male	107 (70 %)
Ischaemic cardiomyopathy	79 (52 %)
ACE-I	132 (87 %)
Beta-blocker	123 (81 %)
Diuretics	112 (74 %)
NYHA class I/II/III/IV	3/28/121/0 (2/18/80/0 %)
CMR EDV (ml)	283 ± 96
CMR ESV (ml)	217 ± 94
CMR SV (ml)	66 ± 23
CMR EF (%)	24.9 ± 9.6
2D echocardiographic EDV (ml)	213 ± 74
2D echocardiographic ESV (ml)	149 ± 65
2D echocardiographic SV (ml)	64 ± 22
2D echocardiographic EF (%)	31.5 ± 8.7

*CMR* cardiac magnetic resonance imaging, *EF* ejection fraction, *EDV* end-diastolic volume, *ESV* end-systolic volume, *SV* stroke volume

### Left ventricular volumes

**Fig. 1:**
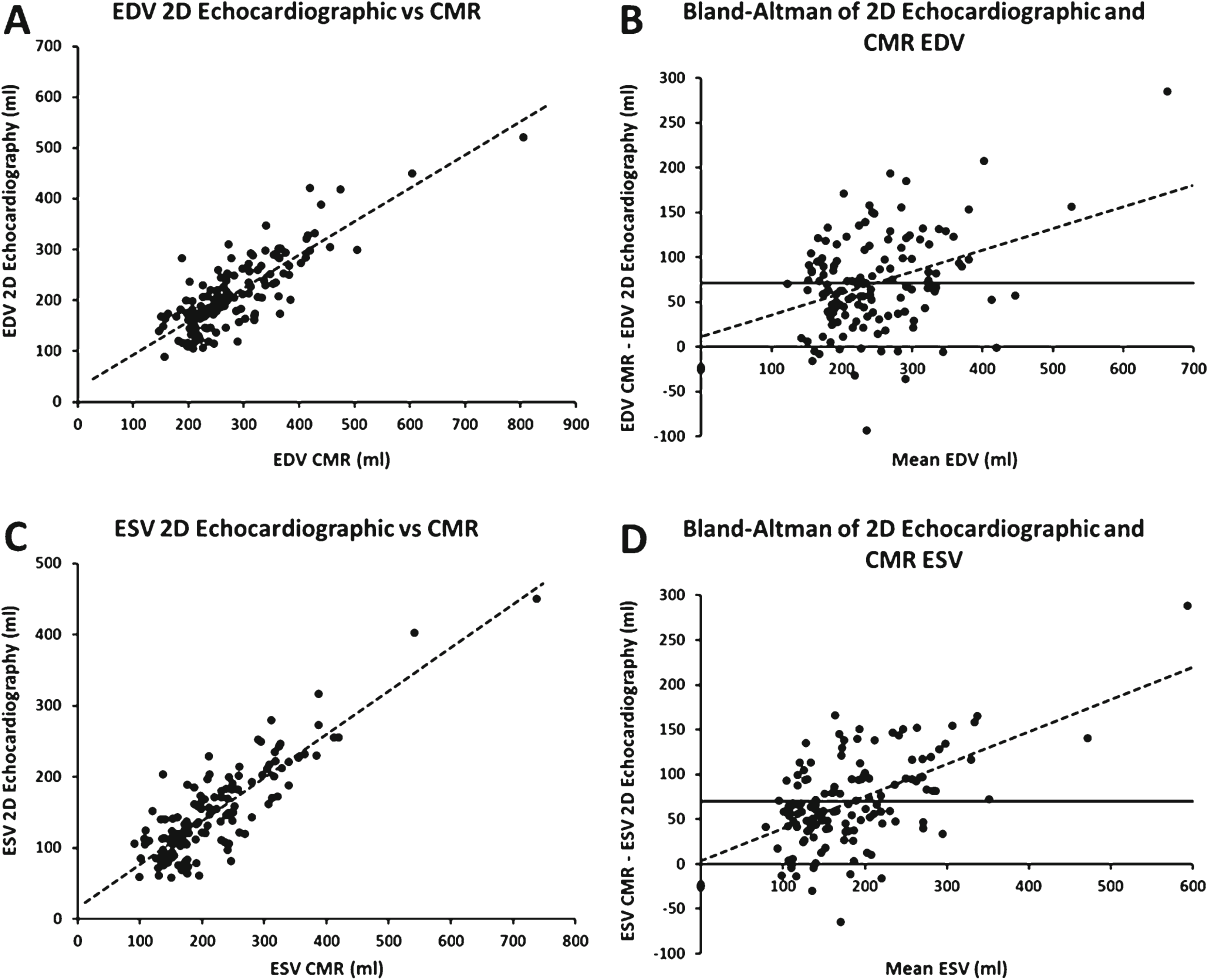
2D echocardiographic and CMR measurements with linear regression line (dotted line) for EDV (**a**) and ESV (**c**). Blant-Altman analysis of EDV (**b**) and ESV (**d**) assessed by 2D echocardiography and CMR, including linear regression line (dotted line). The thick line indicates the mean differenced. CMR: cardiac magnetic resonance imaging; EDV: end-diastolic volume; and ESV: end-systolic volume

EDV and ESV were found to be substantially smaller with 2D echocardiography compared with CMR (Table 1, Fig. 1 panel A and C). On average, 2D echocardiography significantly underestimated EDV and ESV by 71 ± 53 ml and 70 ± 49 ml, respectively. Stroke volumes, however, were comparable between the two techniques. Differences between the imaging modalities increased significantly with increasing left ventricular volumes for both EDV and ESV, as indicated by the regression lines in Fig. 1 (panel B and D), *p* < 0.001 for both. In contrast, the absolute difference (in ml) between echocardiographic and CMR assessment of EDV approximately equalled the absolute difference (in ml) of ESV, independent of volume. When comparing patients with ischaemic cardiomyopathy and dilated cardiomyopathy, the differences in left ventricular volumes between the two imaging modalities were similar (EDV: 65 ± 47 ml and 78 ± 59 ml; ESV: 64 ± 45 ml and 76 ± 54 ml; respectively).

**Fig. 2:**
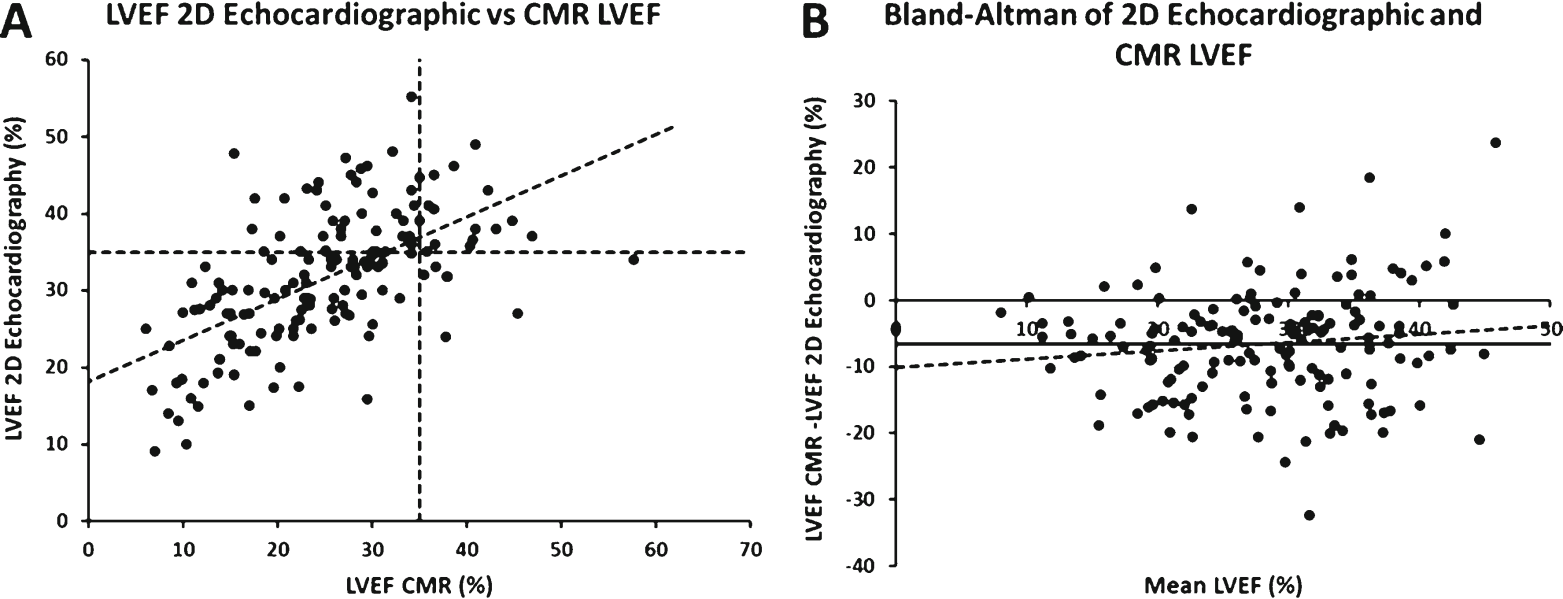
LVEF assessed by 2D echocardiographic and CMR with linear regression line and lines indicating the LVEF cut-off of 35 %, according to the guidelines (**a**). Blant-Altman analysis LVEF by 2D echocardiography and CMR, including linear regression line (**b**). The thick line indicates the mean difference. CMR: cardiac magnetic resonance imaging; and LVEF: left ventricular ejection fraction

Mean LVEF assessed by CMR examination was 24.9 ± 9.6 % and the mean LVEF assessed by 2D echocardiography was 31.5 ± 8.7 %. Blant-Altman analysis showed a consistent overestimation of the LVEF by 2D echocardiography compared with CMR (6.6 ± 8.3 %), with a trend to increase with decreasing LVEF (*p* = 0.161) (Fig. 2). The overestimation of LVEF by 2D echocardiography was similar between the patients with ischaemic cardiomyopathy and those with dilated cardiomyopathy (6.8 ± 8.3 ml and 6.4 ± 8.1 ml; respectively).

### Interobserver and intraobserver variability

Analysis of intraobserver and interobserver variability of the imaging modalities revealed a mean difference of 1.4 ± 7.6 % for the intraobserver variability of the 2D echocardiographic LVEF and a mean difference of 1.5 ± 6.7 % for the interobserver variability of the 2D echocardiographic LVEF. The mean difference for the intraobserver variability of LVEF assessed by the CMR was 0.3 ± 2.7 % and the interobserver variability showed a mean difference of 1.1 ± 3.5 %.

### Consequences for cardiac device eligibility

In the present study population, a significantly different population is selected for device therapy depending on the imaging modality used (*p* < 0.01) (Fig. 2), considering an LVEF cut-off value of 35 %. A substantial proportion of patients (28 %) had opposing outcomes of eligibility for cardiac device therapy depending on the imaging modality used (Table 2). Of the patients, 6 % had an LVEF below 35 % with 2D echocardiography, but above 35 % with CMR and 22 % patients had an LVEF above 35 % with 2D echocardiography, but below 35 % with CMR. In comparison, with 2D echocardiography, 24 additional patients (23 %) would be eligible for cardiac device therapy using CMR.

**Table 2:** Eligibility for cardiac device therapy according to imaging modality

	CMR EF > 35 %	CMR EF ≤ 35 %	Total
Echo EF > 35 %	14 (9 %)	33 (22 %)	47 (31 %)
Echo EF ≤ 35 %	9 (6 %)	96 (63 %)	105 (69 %)
Total	23 (15 %)	129 (85 %)	152 (100 %)

*EF* ejection fraction, *CMR* cardiac magnetic resonance imaging

### Additional findings on CMR

A left ventricular thrombus was seen in 12 patients (7.9 %) by CMR, while only 2 of them (1.3 %) were seen by echocardiography. Therefore, a left ventricular thrombus was not identified by echocardiography in 10 patients (6.6 %).

## Discussion

Present study shows that assessment of left ventricular volume and LVEF by 2D echocardiography and CMR are not interchangeable in a heart failure population and that there is a rather large discrepancy between the two methods. Two-dimensional echocardiography underestimates the left ventricular volumes and overestimates LVEF compared with CMR. The underestimation of left ventricular volumes is larger when these volumes are larger. Differences in LVEF tend to be larger when the LVEF is smaller.

### Left ventricular volumes

Several studies compared 2D echocardiography and CMR for assessment of left ventricular volumes and LVEF.[5, 11–14] These studies differ in number of included patients, aetiology of heart disease and left ventricular function and volume, but all consistently show underestimation of EDV and ESV by 2D echocardiography compared with CMR. Reported mean differences vary considerably between studies and range from 10 to 94 ml for ESV and from 11 to 131 for EDV.[5, 19] We found the discrepancy between the two imaging modalities to be strongly related to left ventricular volume, with increasing differences in larger hearts. This finding is supported by a smaller study by Gruszczynska et al. in ischaemic heart failure patients.[13] It is further corroborated by the observation that studies in hearts with predominantly (near) normal volumes report lower differences, whereas studies including dilated hearts are at the higher end of the spectrum.[5, 11, 14, 20]

The underestimation of left ventricular volumes in 2D echocardiography compared with CMR might be explained by two major factors. Firstly, CMR analysis includes the trabecularisation in the left ventricular cavity, whereas 2D echocardiography does this to a lesser extent. Secondly, in 2D echocardiography both suboptimal transducer position causing foreshortening and gain-dependent edge identification might cause volume underestimation. Several other factors might add to the discrepancy. CMR uses the summation of disks method. At the basal level, the short-axis image typically transects the mitral valve plane and as a consequence the left ventricular cavity might be difficult to determine exactly. On the other hand, Simpson’s biplane method used in 2D echocardiography volume assessment makes geometric assumptions which might not hold true in case of regional deformation, which is present in a substantial number of heart failure patients. These regional deformations are more often seen in patients with ischaemic cardiomyopathy, but the current study did not demonstrate a difference between patients with ischaemic cardiomyopathy and patients with dilated cardiomyopathy.

### Left ventricular ejection fraction

Studies on differences in LVEF assessed by the two imaging techniques reported contradictory results, from an overestimation of 7 % to an underestimation of 4 % by 2D echocardiography compared with CMR.[11, 12] Only two previous studies focused on patients with a severely depressed ejection fraction. In a small study (*n* = 36) Bellenger et al. found no significant difference, but a larger (*n* = 67) more recent study in an ischaemic cardiomyopathy population with low ejection fraction showed an LVEF overestimation of 5.7 % by echocardiography.[5, 13] Our results closely resemble those of Gruszczynska et al. in a larger study population including non-ischaemic cardiomyopathy patients as well.

The inconsistencies among studies in reported differences of LVEF assessment might in part be attributable to differences in study population. From a theoretical point of view, systematic underestimation to a similar extent in EDV and ESV by echo, as observed in several studies including the present one, will result in overestimation of LVEF. This notion is supported by the trend of increasing discrepancy with decreasing LVEF, found in both our study and that of Gruszczynska et al.[13] Moreover, in a study including a wider range of LVEF, Duncan et al. reported a significantly larger echocardiographic overestimation of LVEF with lower LVEF.[21]

LVEF assessment by CMR is generally considered the golden standard. However, data on intraobserver and interobserver variability of LVEF assessed by CMR and 2D echocardiography in heart failure patients are scarce. Some reports show a small increase in observer variability for heart failure patients compared with normal subjects.[22, 23] The present study demonstrated superior intraobserver and interobserver variability of LVEF assessment for CMR compared with 2D echocardiography, comparable with previous studies.[20–22]

### Additional CMR and echocardiography parameters

Left ventricular thrombus is one of the additional findings which can been shown by CMR. In the current study 7.9 % of patients had a left ventricular thrombus on CMR, which is comparable with earlier studies.[15] Conventional echocardiography only discovered 1.3 % of the left ventricular thrombi, which is in line with the known low sensitivity of thrombus detection by echocardiography.[24] Most of these left ventricular thrombi are probably not clinically relevant, as the rate of embolisation is thought to be low.[25] However, testing of the defibrillation threshold might be avoided in patients with a left ventricular thrombus, because of the supposed risk of embolisation.

In echocardiography the development of 3D imaging has been interesting. Meta-analysis of multiple studies has shown good correlations between left ventricular volumes assessed by 3D echocardiography and CMR.[26] Although 3D echocardiography underestimates the volumes, LVEF measurements are similar to the measurements obtained by CMR.[26] Moreover, the same results between 3D echocardiography and CMR have been seen in heart failure patients.[27] Therefore, 3D echocardiography might be an appropriate alternative instead of CMR in the assessment of LVEF in patients eligible for device therapy.

In the evaluation for ICD implantation, the LVEF is currently the most important imaging parameter. In the evaluation for CRT the assessment of left ventricular dyssynchrony is an additional aspect next to the LVEF, which can be assessed by both CMR and echocardiography. CMR tagging is capable of measuring dyssynchrony and predicting response.[28] Tissue Doppler imaging and speckle tracking are echocardiographic techniques to assess left ventricular dyssynchrony. Both techniques can predict response to CRT.[29, 30] The assessment of left ventricular dyssynchrony has not yet been incorporated in the guidelines for CRT, but in individual patients it can be of importance.

### Consequences for cardiac device eligibility

The observed differences in assessment of LVEF between the two imaging modalities have significant clinical consequences. Applying the 35 % cut-off value, eligibility for cardiac device therapy will largely depend on the imaging modality used. A substantial proportion of patients (28 %) had opposing outcomes of eligibility for cardiac device therapy when their LVEF was assessed with CMR compared with echocardiography. This was previously shown in two smaller studies that demonstrated that a significant proportion of patients (21 %) were reclassified according to the imaging modality.[15, 16]

Furthermore, in comparison with 2D echocardiography 23 % more patients would be eligible for cardiac device therapy when using LVEF assessed by CMR. This increase in the number of eligible patients was also established in previous research.[15] This rise in eligible patients questions whether one should use the same cut-off value in 2D echocardiography and CMR. A lower cut-off value for LVEF assessed by CMR might be more appropriate in order to select patients most at risk for ventricular arrhythmias; however it could also be assumed that a higher cut-off for 2D echocardiography should be used.

One study has evaluated the impact of CMR-assessed LVEF on ICD therapy up till now.[16] It showed that LVEF assessed by CMR was a better predictor of device therapy compared with LVEF assessed by 2D echocardiography. However, larger studies should reveal the actual impact of CMR and eligibility for device therapy.

### Limitations

Assessments by CMR and echocardiography were not performed simultaneously in the small subset of patients and ultrasound contrast was not used to enhance endocardial contours in any of the patients. Although patients were clinically stable between the two assessments, subclinical changes might have occurred in their condition affecting study outcome. Furthermore, the study was conducted retrospectively and only patients who were referred for device therapy were analysed, which might have introduced a selection bias. However, since this study describes a straightforward comparison of imaging data and not patient outcome, a selection bias is of lesser relevance. Another bias was probably introduced by the echocardiographic image quality. In the current study all patients had a moderate to good echocardiographic image quality and all images were sufficient for volume assessment. Therefore, patients with poor echocardiographic image quality might not be subject to echocardiography prior to the CMR assessment, because of their known poor image quality.

## Conclusion

In conclusion, this study showed a systematic overestimation of LVEF assessed by 2D echocardiography compared with CMR in heart failure patients with a severely depressed LVEF. This implies that the two imaging modalities are not interchangeable in this patient population. The discrepancy may significantly impact clinical decisions for individual patients, for example eligibility for device therapy. As CMR has better observer variability, it would be the preferred method for LVEF assessment in heart failure patients. However, LVEFs assessed by CMR are consistently smaller than those assessed by 2D echocardiography. The present study and previous work point towards the necessity for resetting cut-off values when CMR is used for LVEF assessment. Future studies should address this necessity and should evaluate whether LVEF assessed by CMR improves prediction of beneficial effects of device therapy compared with echocardiographic evaluation.